# The molecular determinants of antigenic drift in a novel avian influenza A (H9N2) variant virus

**DOI:** 10.1186/s12985-022-01755-9

**Published:** 2022-02-05

**Authors:** Yiqing Zheng, Yanna Guo, Yingfei Li, Bing Liang, Xiaoyuan Sun, Shijia Li, Huizhi Xia, Jihui Ping

**Affiliations:** 1 MOE Joint International Research Laboratory of Animal Health and Food Safety, Engineering Laboratory of Animal Immunity of Jiangsu Province, College of Veterinary Medicine, Nanjing Agricultural University, Nanjing, 210095 China; 2 School of Industrial Biotechnology, Jiangnan University, Wuxi, 214122 China

**Keywords:** Avian influenza virus, H9N2, Hemagglutinin (HA) gene, Antigenic drift, Antigenic map

## Abstract

**Background:**

In early 2020, a novel H9N2 AIV immune escape variant emerged in South China and rapidly spread throughout mainland China. The effectiveness of the current H9N2 vaccine is being challenged by emerging immune escape strains. Assessing key amino acid substitutions that contribute to antigenic drift and immune escape in the HA gene of circulating strains is critical for understanding virus evolution and in selecting more effective vaccine components.

**Methods:**

In this study, a representative immune escape strain, A/chicken/Fujian/11/2020 (FJ/20), differed from current H9N2 vaccine strain, A/chicken/Anhui/LH99/2017 (AH/17) by 18 amino acids in the head domain in HA protein. To investigate the molecular determinants of antigenic drift of FJ/20, a panel of mutants were generated by reverse genetics including specific amino acids changes in the HA genes of FJ/20 and AH/17. The antigenic effect of the substitutions was evaluated by hemagglutination inhibition (HI) assay and antigenic cartography.

**Results:**

Fujian-like H9N2 viruses had changed antigenicity significantly, having mutated into an antigenically distinct sub-clade. Relative to the titers of the vaccine virus AH/17, the escape strain FJ/20 saw a 16-fold reduction in HI titer against antiserum elicited by AH/17. Our results showed that seven residue substitutions (D127S, G135D, N145T, R146Q, D179T, R182T and T183N) near the HA receptor binding sites were critical for converting the antigenicity of both AH/17 and FJ/20. Especially, the combined mutations 127D, 135G, 145N, and 146R could be a major factor of antigenic drift in the current immune escape variant FJ/20. The avian influenza A (H9N2) variant virus need further ongoing epidemiological surveillance.

**Conclusions:**

In this study, we evaluated the relative contributions of different combinations of amino acid substitutions in the HA globular head domain of the immune escape strain FJ/20 and the vaccine strain AH/17. Our study provides more insights into the molecular mechanism of the antigenic drift of the H9N2 AIV immune escape strain. This work identified important markers for understanding H9N2 AIV evolution as well as for improving vaccine development and control strategies in poultry.

**Supplementary Information:**

The online version contains supplementary material available at 10.1186/s12985-022-01755-9.

## Background

In recent year, novel avian influenza viruses (AIVs) have emerged as a major threat to animal and human [1]. The focus of global pandemic preparedness is H5 and H7 AIVs [2, 3]. However, the pandemic threat posed by other subtypes of avian influenza viruses, especially the H9 subtype, should not be overlooked. H9N2 AIVs are widely spread in poultry worldwide and causes severe economic losses to poultry industries [4]. H9N2 AIV has gradually become the most prevalent subtype of AIVs in mainland China since 1994 [5, 6]. Available evidence has demonstrated that H9N2 viruses isolated naturally from poultry have acquired the ability to preferentially bind to the human-type receptor [7, 8]. Due to sporadic cases of human infection, and by providing reassortant internal genes to human-infecting subtypes such as H5N1, H5N6, H7N9, and H10N8 [9–12], H9N2 AIV is posing a significant threat to public health with potential pandemic risk. Although conventional inactivated vaccines were used in many countries for disease control, the virus continues to circulate in vaccinated chicken farms, which is possibly due to antigenic drift [13]. Antigenic drift is mediated by the gradual accumulation of mutations in haemagglutinin (HA) glycoprotein, which results in viruses that escape from prior antibodies produced by natural infection or vaccination. Therefore, HA is the focus of influenza virus surveillance and a major component of currently licensed vaccines. Understanding the antigenic property of circulating viruses is essential for updating matched vaccine strains to control the circulation of H9N2 in poultry.

In previous studies, a certain number of escape mutations in the HA gene of H9N2 virus have been confirmed by using a common monoclonal antibodies (mAbs) escape mutant method [14–16], and several residues were assigned to two discrete antigenic sites, ‘H9-A’ and ‘H9-B antigenic epitopes [17]. These residues identified by mAbs were important for understanding the viral antigenicity; however, many residues are completely conserved among circulating viruses. While one or two amino acid changes can cause sufficient antigenic drift in some situations, simultaneous substitutions linked with unselected mutations at several sites is most generally observed [18–20]. Hence, an improved understanding of the molecular basis of antigenic differences between two related strains could ultimately facilitate the selection of more effective vaccine components to control the circulation of influenza in poultry.

The novel H9N2 AIV immune escape variant had been circulating in Fujian province, China since early 2020. The current H9N2 vaccine strain did not antigenically match the circulating Fujian-like viruses, which caused a H9N2 outbreak across almost the entire country soon afterwards. In this study, we selected a representative antigenic escape strain A/chicken/Fujian/11/2020 (FJ/20) and the vaccine strain A/chicken/Anhui/LH99/2017 (AH/17) as models through standard Hemagglutination inhibition (HI) assays and antigenic cartography to explore the molecular determinant of antigenic variation of FJ/20. The 18 sites in the globular head domain of HA, sites where FJ/20 and AH/17 expose different amino acids, were combined into groups according to their proximity on the membrane-distal surface of the HA protein. Subsequently, the specific amino acid combinations were introduced into the two HA genes and all viruses were generated by reverse genetics in A/PR/8/34 (PR/8, H1N1) backbone. The HI assay, with relevant monovalent chicken antisera, and antigenic cartography were employed to identify the minimal amino acid substitutions were responsible for the immune evasion of FJ/20. To better understand the evolution, variability and relative prevalence of key antigenic sites of influenza A(H9N2) in China, additional bioinformatics analysis of the published H9 sequences was also performed.

Our study elucidated the antigenic effects of specific amino acid substitutions in a novel H9N2 AIV immune escape variant, and provided an improved understanding of the antigenic drift and evolution of circulating H9N2 influenza viruses. Our work will facilitate a rational approach for selecting matched vaccine strains to control the circulation of influenza in poultry and to reduce the potential for zoonotic viruses to emerge.

## Methods

### Viruses and cells

The following six H9N2 vaccine strains and one immune escape representative strain were used in this study: A/chicken/Henan/HP/1998 (HN/98), A/chicken/Shandong/6/1996 (SD/96), A/chicken/Guangxi/55/2005 (GX/05), A/chicken/Jiangsu/WJ57/2012 (JS/12), A/chicken/Jiangsu/325/2018 (JS/18), A/chicken/Anhui/LH99/2017 (AH/17), and escape representative A/chicken/Fujian/11/2020 (FJ/20).

The immune escape strain A/chicken/Fujian/11/2020 (FJ/20) was a representative antigenic escape strain, isolated from vaccinated chickens in Fujian province of southeast China in 2020, which was collected as samples of routinely ongoing surveillance. The viral subtype was identified by a standard Hemagglutination inhibition (HI) test and reverse-transcription polymerase-chain reaction (RT-PCR). The virus was purified via three rounds limiting dilution in 9–11-day specific-pathogen-free (SPF) embryonated chicken eggs. And the entire genome of FJ/20 was amplified by RT-PCR using segment-specific primers and confirmed by Sanger sequencing.

Human embryonic kidney (293 T) cells were maintained in Dulbecco’s modified Eagle medium (DMEM) supplemented with 10% fetal calf serum (Biological Industries), penicillin (100 U/ml), and streptomycin (100 μg/ml) at 37 °C with 5% CO_2_.

### Phylogenetic analysis

The published HA sequences of Chinese H9N2 influenza A virus (2017–2019) and reference strains used in phylogenetic analysis were acquired from GISAID database (https://www.gisaid.org).

The 15 classical H9N2 reference strains were as following: A/turkey/Wisconsin/1/1966, A/chicken/Heilongjiang/35/00, A/shorebird/Delaware/9/96, A/quail/Arkansas/29209-1/93, A/duck/Hong Kong/Y439/97, A/chicken/Korea/38349-p96323/96, A/quail/Hong Kong/G1/97, A/chicken/Henan/2/98, A/duck/Hong Kong/Y280/97, A/chicken/Shanghai/B469/2011, A/chicken/Guangdong/FZH/2011, A/chicken/Shanghai/F/98, A/chicken/Guangxi/55/2005, A/chicken/Shandong/6/96, A/chicken/Guangxi/55/2005. GenBank accession numbers for all analyzed sequences are listed in Additional file 1: Table S1.

The resulting sequences were aligned by using MAFFT version 7 [21], manually adjusted to correct frame-shift errors, and subsequently translated. The Maximum likelihood (ML) tree was constructed with the MEGA (7.0) software [22] under the Jones-Taylor-Thornton (JTT) model with 1000 bootstraps. The ML tree was visualized alongside amino-acid alignments using Evolview2.0 [23].

A time-scaled phylogeny of H9N2 HA was analyzed using the Nextstrain platform [24] (https://github.com/nextstrain/avian-flu). The HA sequences of avian H9N2 in China from 1994 to 2020 were obtained from GISAID database (https://www.gisaid.org). Excluding homologous and duplicate sequences, a total of 6454 sequences were used for data analysis. In brief, the sequences were aligned by MAFFT version 7 [21], a phylogeny was constructed using IQTree [25] using a generalized time-reversible (GTR) model and a time-scaled phylogeny was inferred using TreeTime [26]. To facilitate discussion of genetic diversity, we identified sequences in different clusters on the basis of the presence of amino acid substitutions. And then the HA sequences without definite sampling locations from the above 6454 sequences were removed, the filtered 6201 sequences in the subtrees were counted towards the geographic distribution represented in the pie charts in extended Data (Fig. 7).

### Generation of 6:2 recombinant viruses by reverse genetics

The HA and NA genes of AH/17 and FJ/20 were cloned into vRNA expressing vector, pHH21. The specific amino acid combinations were introduced into the AH/17 and FJ/20 HA by PCR-based site-directed mutagenesis. Primer sequences are available upon request. All the constructs were completely sequenced to ensure the absence of unwanted mutations.

The recombinant viruses were generated in the A/Puerto Rico/8/34 (PR8, H1N1) backbone, harboring the HA and NA genes of AH/17 or FJ/20, as previously described by the “twelve-plasmid” reverse genetics system [27]. Briefly, 1 µg of each of the four protein-expressing plasmids and 0.2 µg of each of the vRNA expressing plasmids transfected into 70–80% confluent 293 T cells using Lipofectamine™ 2000 Transfection Reagent (Invitrogen, Carlsbad, CA, USA). After incubation for 6 h at 37 °C, the transfection mixture was replaced by Opti-MEM (Gibco, USA) and 24 h later 0.5 μg/ml TPCK-treated trypsin were added. At 48 h post-transfection, the cell supernatants were inoculated into 10-day-old SPF embryonated chicken eggs for virus propagation. The HA gene of rescued viruses was sequenced to verify that additional mutations did not arise.

### Generation of H9N2 virus mono-specific antiserum

The allantoic fluid containing different strains was inactivated by adding 0.2% formalin(v/v) for 24 h at 37 °C. Inactivation was confirmed by the absence of detectable infectivity after two blind passages of formalin-treated allantoic fluid in embryonated eggs. The inactive allantoic fluid was mixed with Tween-80 in a homogenizer and then emulsified with two parts of commercial white oil (v/v). The mono-specific antisera of reference viruses and mutated viruses were generated by using six-week-old SPF chickens, two chicken per group. Each chicken was injected with 500 μL of oil emulsion inactivated vaccine. Chicken blood was collected at 21 days after inoculation to separate the mono-specific anti-sera and the antisera was stored at − 20 °C. The information of mono-specific anti-sera generated in this study is listed in Additional file 1: Table S2.

### HI assay and antigenic cartography

The hemagglutination inhibition (HI) assay was performed in 96-well microtiter plates as previously described [28], with an initial serum dilution of 1:2. Briefly, each serum sample was serially diluted and added to 8 hemagglutination units (HAU) of each recombinant virus. After incubation for 30 min at room temperature, 1% chicken erythrocytes were added to all wells and the plate was gently agitated to mix. Agglutination was read out after incubation for 30–45 min at room temperature. The HI titer was expressed as the reciprocal of the highest antibody dilution that completely inhibited hemagglutination. And then the HI titers are mathematically transformed to generate a table of antigen distances that were used for antigenic cartography.

Antigenic cartography is a computational method that enables high-resolution quantitative analyses and visualizations of HI assay data, and was previously used for Human (H3), swine (H3), equine (H3), and avian (H7) influenza A virus [29–32]. The antigenic map quantifies and displays antigenic differences as interrelated distances between viruses. The distance between viruses is measured by antigenic units (AU), and 1 AU is equivalent to a 2-fold dilution in the HI assay. Antigenic maps of the viruses were obtained by using Antigenic Cartography software (http://www.antigenic-cartography.org/).

### Structural and data analysis

The 3D structure of the FJ/20 HA protein was obtained via homology modeling, by using SWISS-MODEL [33]. The Amino acid positions were plotted on the surface of HA molecules using Pymol software (Schrödinger).

For bioinformatic analysis, all H9 HA amino acid sequences of China (as of November 2019) were downloaded from GISAID database (https://www.gisaid.org) and aligned using MAFFT version 7 [21]. The distribution of amino acids at each antigenic residue was then assessed.

## Results

### Molecular characters and phylogenetic analyses

**Fig. 1:**
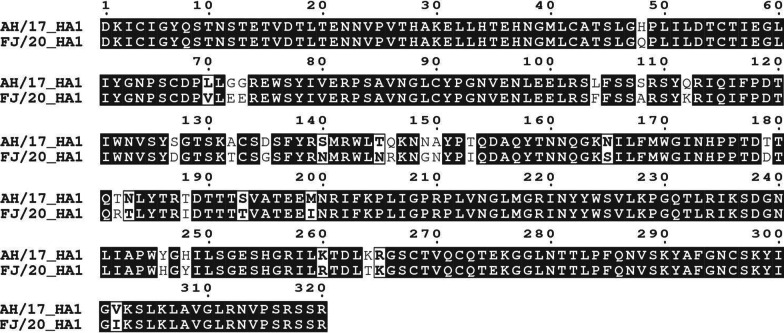
The amino acid differences between FJ/20 and vaccine strain AH/17 in the HA1 protein

**Fig. 2:**
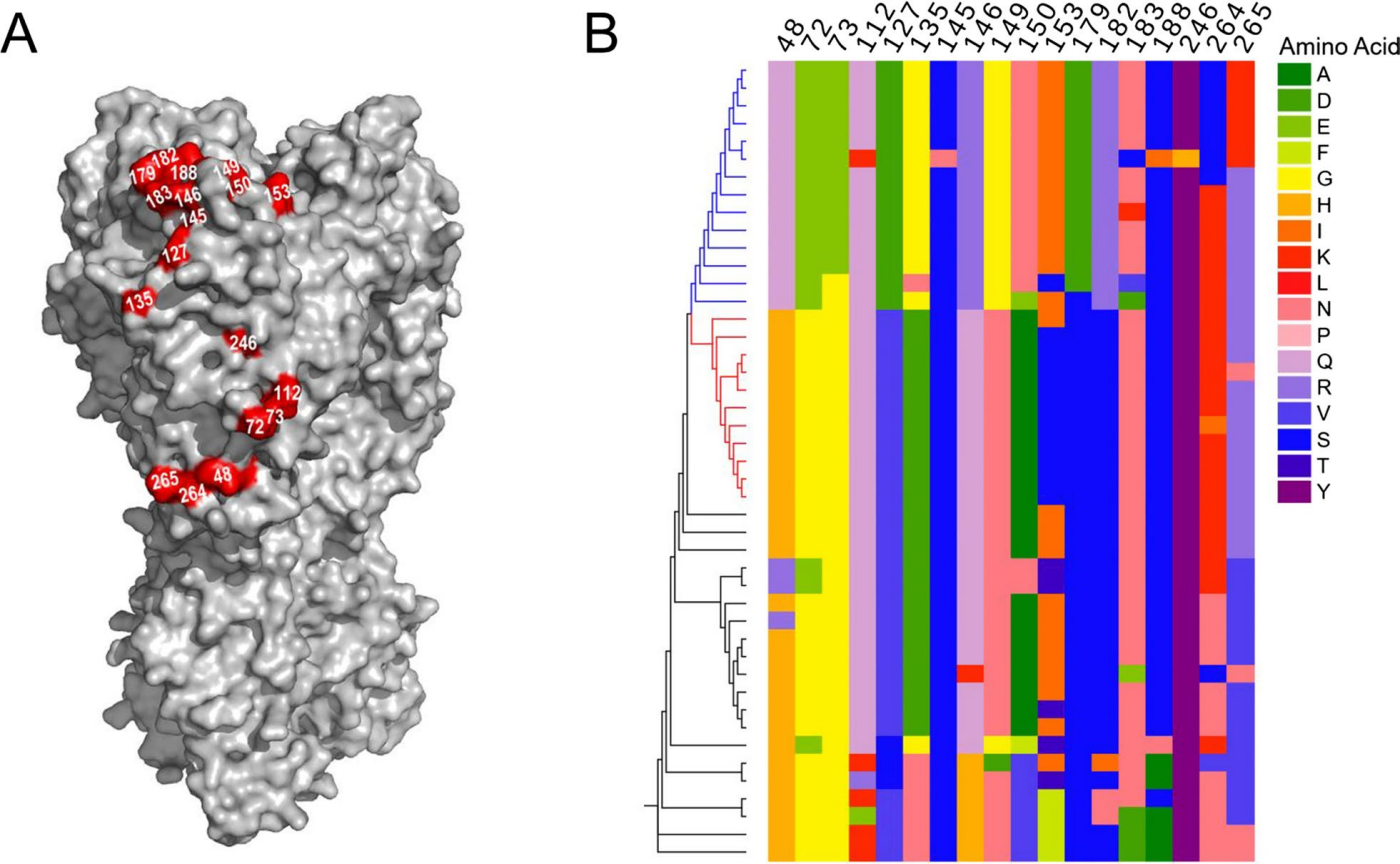
Sequence variation at the mutant sites in HA1 of H9N2 influenza A viruses. **A** Homotrimers of FJ/20 HA showing the amino acid difference between FJ/20 and AH/17. The mutations are shown as red with H9 numbering. **B** Maximum likelihood (ML) phylogeny of the H9N2 influenza virus HA amino acid sequence. The HA gene sequences from reference H9N2 viruses were downloaded from GISAID’s EpiFlu Database. The ML tree was constructed with the MEGA (7.0) software under the Jones–Taylor–Thornton (JTT) model with 1000 bootstraps. Vaccine strain AH/17 is in the red sub-clade. The blue sub-clade contains FJ/20. Amino-acid identity is shown by color, grouped by side-chain property, according to the legend

Since 2017, we have contentious monitored and isolated avian H9N2 IAVs from live poultry markets and chicken farms. On the early 2020, we isolated one H9N2 virus, named as A/chicken/Fujian/11/2020 (FJ/20) from South China, and found that it had a significantly different antigenicity with previous H9N2 AIVs. After then, a large number of Fujian/20-like H9N2 viruses was identified from the clinical samples in South China, East China and many other provinces. Therefore, in this study, Fujian/20-like representative virus A/chicken/Fujian/11/2020 (FJ/20) and the current H9N2 vaccine strain A/chicken/Anhui/LH99/2017 (AH/17) were selected as models to reveal the molecular basis of antigenic drift and vaccination failure. The HI assay between AH/17 and FJ/20 revealed a typical pattern of influenza virus antigenic drift, with a 16-fold reduction in HI titers compared to AH/17 homologous antisera (Table 1). After sequencing, the amino acid (AA) sequence of the HA1 region showed 29 residues differences between AH/17 and FJ/20 (Fig. 1). The substitutions on the membrane surface of HA protein globular head domain could be the most responsible for affecting the recognition and neutralization of antibodies produced by previous vaccination, leading to antigenic mismatch and vaccination failure [34]. Therefore, we focused on the 18 AA substitutions located on the HA globular head region (Fig. 2A) to further evaluate their contributions to the evolution and antigenic drift of FJ/20.

**Table 1:** The hemagglutinin inhibition assay (HI) between AH/17 and FJ/20

Virus	HI titer^a^	
	Anti-A/Anhui/LH99/2017	Anti-A/Fujian/11/2020
A/chicken/Anhui/LH99/2017(AH/17)	2048	128
A/chicken/Fujian/11/2020(FJ/20)	128	2048

^a^Chicken vaccinated serum against AH/17 or FJ/20 was tested with the two viral antigens by the HI assay. The HI titer (averaged from two animals for anti-FJ/20) was the reciprocal of the highest serum dilution that inhibited hemagglutination

The genetic variation of the FJ/20 was assessed by maximum likelihood algorithms in an HA gene phylogenetic tree and the amino acid identity at the 18 HA positions was shown by color and grouped by side chain property (Fig. 2B). We found the vaccine strain AH/17 and the immune escape strain FJ/20 belonged to two different sub-clades, colored by red and blue, respectively. In addition, significant differences among most of the 18 sites in the two subclades, such as positions of 48, 73, 74, 127, 135, 146, 149, 150, 153, 179 and 182, suggested that these sites may contribute to virus evolution and immune evasion. Collectively, the above data indicated that Fujian-like H9N2 viruses had changed antigenicity significantly, having mutated into an antigenically distinct sub-clade, which could be responsible for the increasing prevalence of H9N2 outbreaks in vaccinated chickens.

### Chimeric viruses rescue for antigenic analysis

**Fig. 3:**
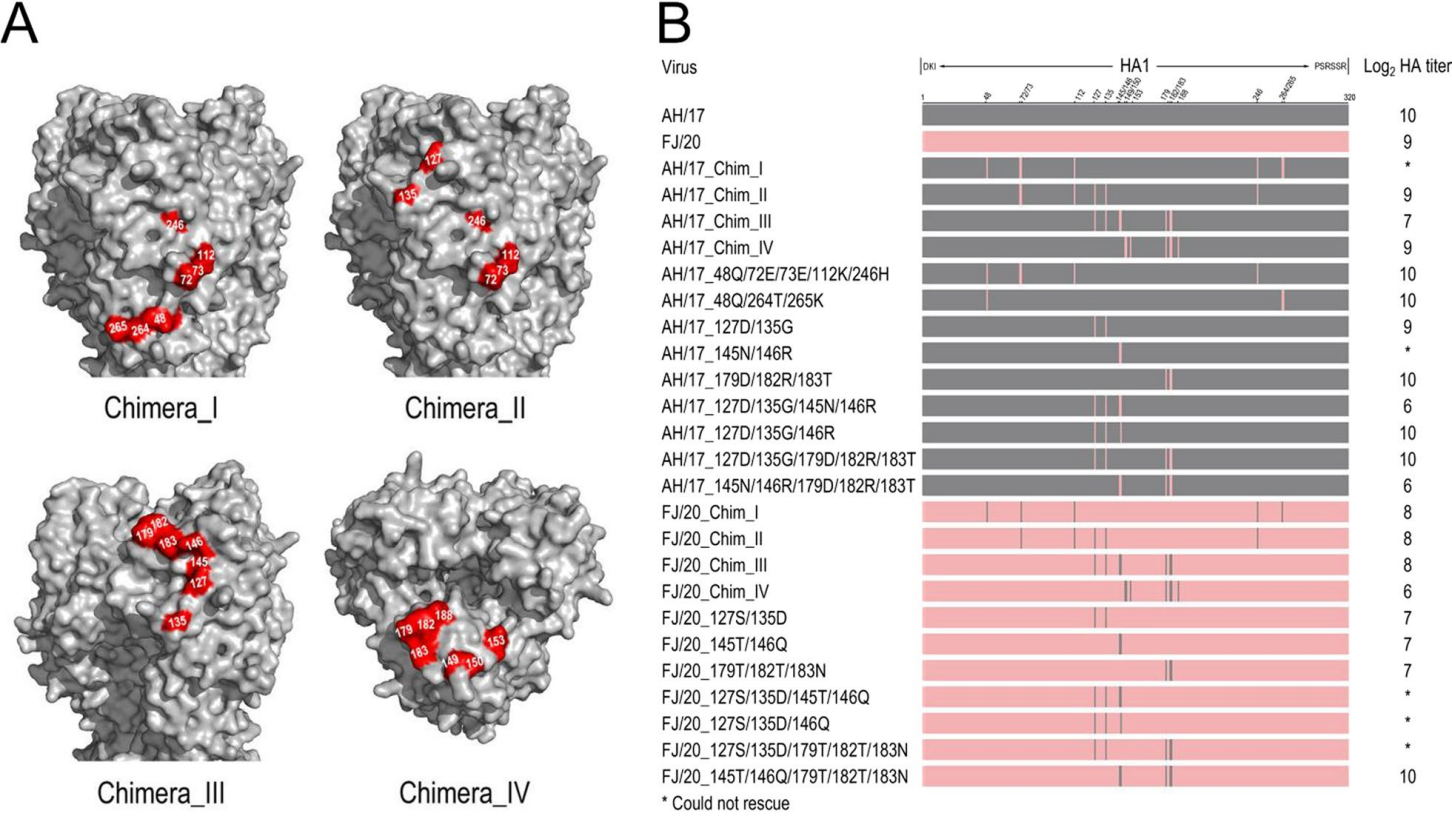
Schematic diagram of AH/17 and FJ/20 chimeras and mutants. **A** Amino acid substitutions in four chimeric viruses. Four types of chimeras were divided and grouped according to the position of different amino acids on the surface of HA protein. **B** Schematic illustration of the rescued chimeras and mutants by reverse genetics. Amino acid substitutions were introduced into the AH/17 and FJ/20 HA genes by PCR-based site-directed mutagenesis and viruses were generated by 6:2 reverse genetics system

To reveal the molecular basis of the antigenic drift and vaccination failure, the targeted 18 AA substitutions were grouped into four combinations according to antigenic epitopes and spatial location, and called them chimeras (Fig. 3A). Each chimera contains 6 to 7 substitutions. To further confirm the key substitutions leading to significant antigenic change observed from a specific chimeric HI result, the multiple AA substitutions were divided into several subgroups and the subgroups were further reorganized into pairs. The set of mutations in each chimeric HA was reciprocally exchanged between the FJ/20 and AH/17 HA genes via PCR-based site-directed mutagenesis. Subsequently, the recombinant viruses were rescued in a backbone of A/Puerto Rico/8/34 (PR8, H1N1), harboring the HA (wild-type, chimeric, or mutant) and NA genes of AH/17 or FJ/20. Unfortunately, with more than three rounds virus rescue attempts, five recombinant viruses could not be generated. In total, twenty-one rescued viruses were used to evaluate the impact of these amino-acid substitutions on the antigenic drift (Fig. 3B).

### The substitutions in the Chimera_III were crucial for FJ/20 antigenic drift

**Fig. 4:**
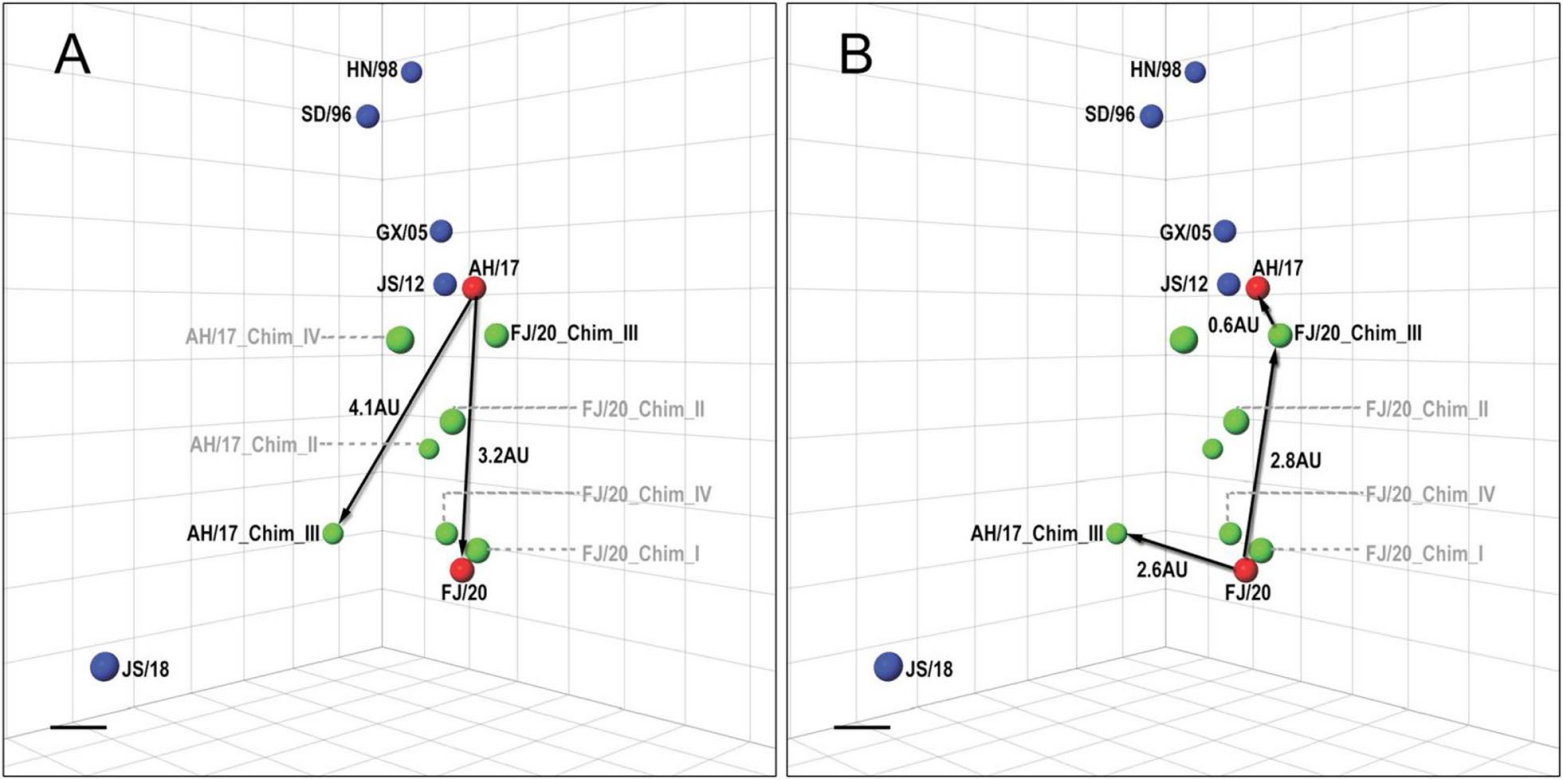
Three-dimensional (3D) antigenic maps of AH/17 and FJ/20 chimeras and reference viruses. The scale bar in each map represents 1 antigenic unit (1 antigenic unit corresponds to a twofold dilution of antiserum in the HI assay). **A** The antigenic distance between AH/17 and FJ/20, AH/17_Chim_III. **B** The antigenic distance between FJ/20 and FJ/20_Chim_III, AH/17_Chim_III, AH/17 and FJ/20_Chim_III

The chimeras, mutants, and reference viruses were examined via HI assay. Mono-specific chicken antisera was produced by immunizing SPF chickens with wild types and mutant viruses. The HI titers were then analyzed using antigenic cartography. The antigenic distances among different viruses on the antigenic map were measured with antigenic units (AU), and 1 AU corresponds to a twofold dilution of antiserum in the HI assay. An antigenic distance of two AUs is generally considered significant and may warrant an update of the human seasonal vaccine strain [35]; therefore, an antigenic distance of 2 AUs can be considered as antigenic difference. The reference vaccine strains and the four pairs of AH/17- and FJ/20-chimeric viruses were shown in the antigenic map (Fig. 4).

The three chimeras of AH/17 had different degrees of antigenic change, with antigenic distances from the AH/17 parent ranging from 2.1 to 4.1 AU (Fig. 4A, Table 2). The mutants AH/17_48Q/72E/73E/112K/246H and AH/17_48Q/264T/265K were generated as a means to simulate the unrescuable AH/17_Chim_I; the relative antigenic positions were shown in Additional file 2: Fig. S1. The changes induced by these two mutants were both around 1.5 AU relative to AH/17, and so they did not strictly reach a significant antigenic threshold (Table 2).

**Table 2:** Antigenic distance information of AH/17, FJ/20 and their mutants

AH/17 and FJ/20 mutants	Distance from AH/17^a^	Distance from FJ/20^a^	Color in the antigenic map^b^
AH/17	–	3.2	Red
FJ/20	3.2	–	Red
AH/17_Chim_II	3.3	3.0	Green
AH/17_Chim_III	4.1	2.8	Green
AH/17_Chim_IV	2.1	3.1	Green
AH/17_48Q/72E/73E/112K/246H^c^	1.5	3.3	Green
AH/17_48Q/264T/265K^c^	1.5	3.6	Green
AH/17_127D/135G	3.4	3.2	Violet
AH/17_179D/182R/183T	2.5	3.5	Violet
AH/17_127D/135G/145N/146R	3.9	2.7	Violet
AH/17_127D/135G/146R	3.6	3.1	Blue
AH/17_127D/135G/179D/182R/183T	2.7	2.5	Orange
AH/17_145N/146R/179D/182R/183T	3.1	3.3	Orange
FJ/20_Chim_I	2.9	0.4	Green
FJ/20_Chim_II	1.8	1.9	Green
FJ/20_Chim_III	0.6	2.6	Green
FJ/20_Chim_IV	3.1	1.2	Green
FJ/20_127S/135D	2.4	1.3	Violet
FJ/20_145T/146Q	2.3	0.9	Violet
FJ/20_179T/182T/183N	3.0	0.6	Violet
FJ/20_145T/146Q/179T/182T/183N	3.0	1.3	Orange

^a^One unit of antigenic distance is equal to a twofold difference in the HI assay

^b^The color indicates the colored dots in Figs. 4 and 5

^c^The positions on the antigenic map were shown in Additional file 2: Fig. S1

The antigenic changes found in the four chimeras of FJ/20 ranged from 0.4 AU to 2.6 AU relative to their parent FJ/20. Chimeras FJ/20_Chim_I and FJ/20_Chim_IV had only minor antigenic changes compared to its parent virus. However, the chimera_II design for both AH/17 and FJ/20 induced a moderate level of antigenic change, with distances of 3.3 and 1.9 AU respectively. This nearly reciprocal result suggests that the substitutions found in the chimera_II design may have significant antigenic impact. Remarkably, the chimera_III design for both AH/17 and of FJ/20 showed the greatest antigenic drift, with distances of 4.1 AU and 2.8 AU relative to their parental viruses (Fig. 4A). Although the antigenic difference between AH/17_Chim_III and FJ/20 is 2.6 AU, the distance between FJ/20_Chim_III and AH/17 is much less, placing it only 0.6 AU away from the wild type AH/17 virus. In this way, FJ/20_Chim_III is now rendered a close match to the current vaccine strain. Thus, the mutations found in the chimera_III design are critical for conferring the antigenic property of AH/17 onto FJ/20 (Fig. 4B).

### Residues at positions 127, 135, 145 and 146 had the greatest impact on antigenic alteration

To further determine which sites were most responsible for the antigenic change, we divided the sites from chimera_III into several sub-groups according to their positions on the surface of the HA protein. The recombinant sub-grouped viruses for both AH/17 and FJ/20 were cloned and rescued (Fig. 3B).

**Fig. 5:**
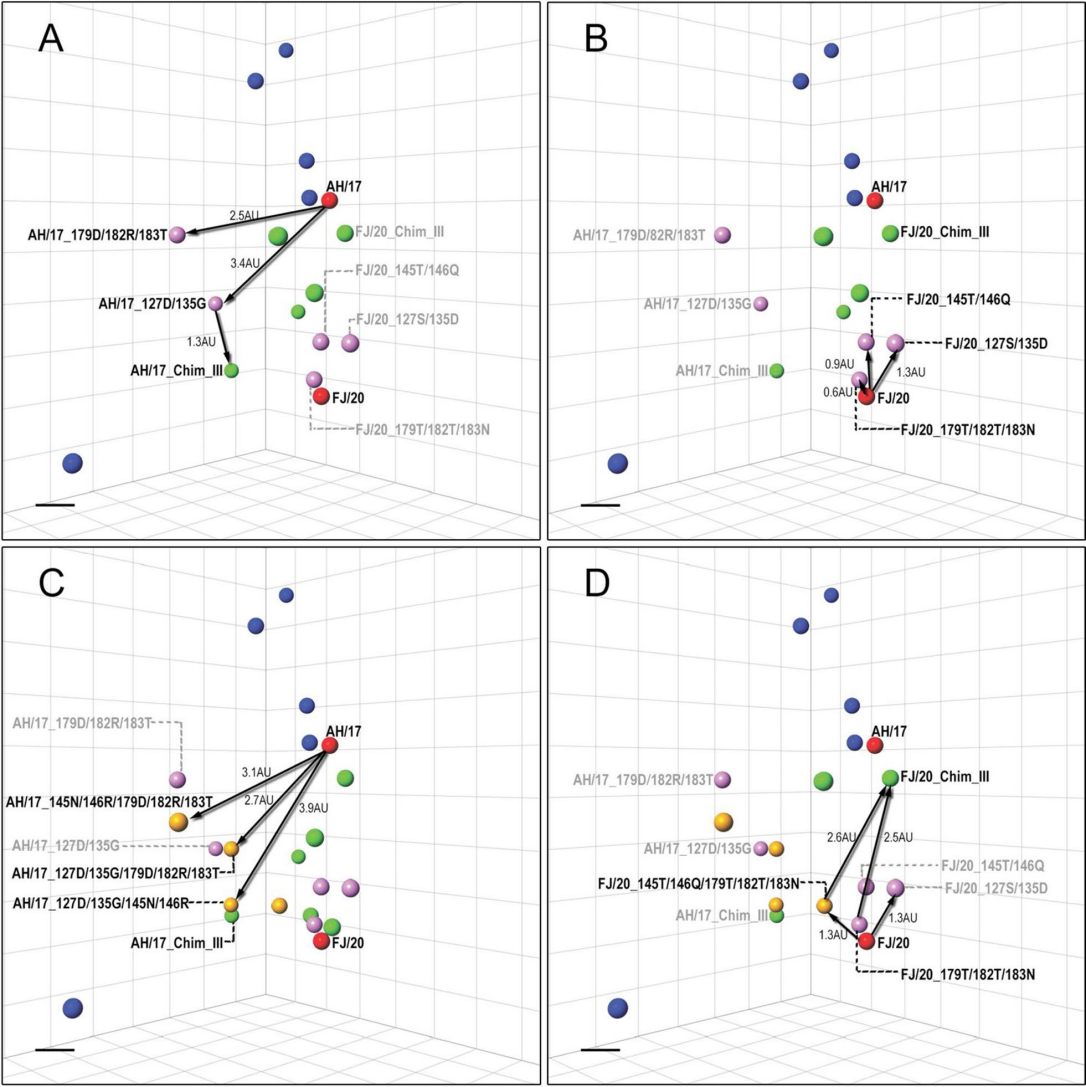
Three-dimensional (3D) antigenic maps of AH/17 and FJ/20 chimeras and mutants. **A** The antigenic map of AH/17, AH/17_127D/135G, AH/17_179D/182R/183T and AH/20_Chim_III. **B** The antigenic map of FJ/20 and its mutants FJ/20_127S/135D, FJ/20_145T/146Q, FJ/20_179T/182T/183N. **C** The antigenic map of AH/17 and its mutants AH/17_127D/135G/145N/146R, AH/17_127D/135G/179D/182R/183T, AH/17_145N/146R/179D/182R/183T. **D** The antigenic map of FJ/20 and FJ/20_145T/146Q/179T/182T/183N

Compared with the other two site combinations, including HA-145/146, or HA-179/182/183 mutations, the recombinant virus carrying 127 and 135 mutations, had the most pronounced effect on the antigenicity whether the backbone was AH/17 or FJ/20 (Table 2). That the chimera_II designs achieved moderate antigenic differences could be seen as consistent with the chimera_III results due to the overlap in their designs at sites 127 and 135. Due to the residue in position 145 being quite conserved, the AH/17_145N/146R might require other compensatory mutations for successful virus rescue. For this reason, the effect of 145N and 146R on antigenicity could not be evaluated at this time. Compared with AH/17_179D/182R/183T, it seemed that the substitutions at position 127 and 135 had a greater impact on antigenicity, achieving a distance between AH/17_127D/135G and AH/17 WT of 3.4 AU (Fig. 5A). Moreover, the proximity of AH/17_127D/135G to AH/17_Chim_III on the antigenic map demonstrates that these two sites contributed much of the distance (Fig. 5A).

The three mutants, FJ/20_127S/135D, FJ/20_145T/146Q and FJ/20_179T/182T/183N drifted from wild type by 1.3, 0.9 and 0.6 AU, respectively (Fig. 5B). Then we evaluated the antigenicity of AH/17_127D/135G/145N/146R, AH/17_127D/135G/179D/182R/183T, AH/17_145N/146R/179D/182R/183T and FJ/20_145T/146Q/179T/182T/183N. Surprisingly, there were two noticeable pairs of viruses whose antigenicity were similar: the first pair being AH/17_127D/135G and AH/17_127D/135G/179D/182R/183T, and the second pair being AH/17_127D/135G/145N/146R and AH/17_Chim_III (Fig. 5C). This indicated that 127D and 135G were the major determinants of antigen change in AH/17_127D/135G/179D/182R/183T. The result also suggested that these four substitutions (127D, 135G, 145N and 146R) have the similar impact on antigenicity as the seven substitutions (127D, 135G, 145N, 146R, 179D, 182R and 183T) found in AH/17_Chim_III. Therefore, the data showed that S127D, D135G, T145N and N146R were the minimal amino acid substitutions that lead to significant changes in antigenic property of AH/17, resulting in a 3.9 AU antigenic change.

When the antigenicity of FJ/20_145T/146R/179T/182T/183R and FJ/20_Chim_III were compared, it was determined that simply substituting two amino acids, S127D and D135G, was sufficient to produce antigenic variants with approximately sixfold difference in HI titer (Fig. 5D). Notably, the four substitutions (S127D, D135G, T145N, N146R) caused a 2.5 AU distance between FJ/20_Chim_III and FJ/20_179T/182T/183R (Fig. 5D); however, that the antigenicity of FJ/20_179T/182T/183R was similar to FJ/20 serves as further support for the idea that these four amino acid substitutions also contributed significantly to antigenic change in FJ/20_Chim_III. These results suggested that the reciprocally substituted chimeric residues of AH/17 and FJ/20 were not equally contributing to the antigenic change. This can be seen where residues exchanged from FJ/20 could induce a more significant impact on AH/17 antigenicity than vice versa. In addition, the simultaneous substitutions of 127D and 135G might contribute significantly to antigenic change. Substitution with only 127D, 135G, 145N, and 146R was sufficient to induce significant antigenic changes in both AH/17 and FJ/20_Chim_III. These four sites could be the major molecular determinants of the antigenic changes in the current immune escape variant FJ/20, and could be related to the evolution of other circulating H9N2 viruses.

### Bioinformatical analysis of the seven residues in chimera_III viruses

**Fig. 6:**
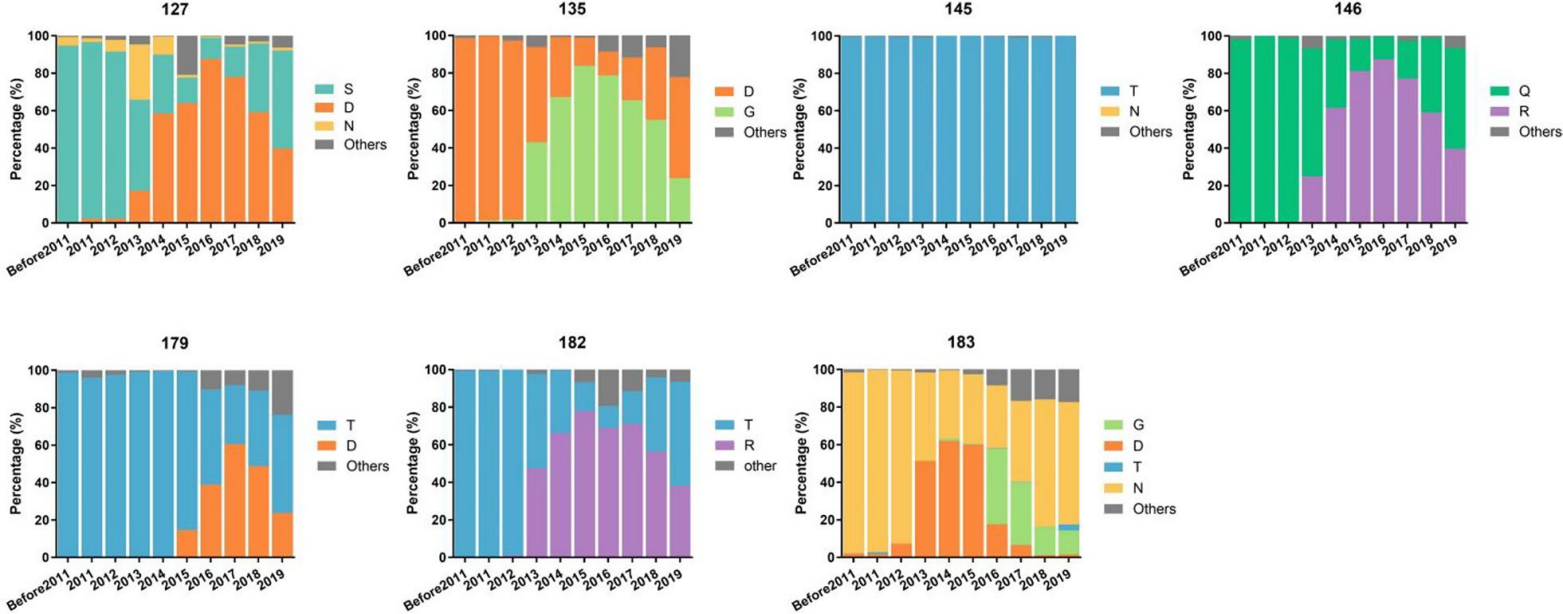
Evolution of residues 127, 135, 145, 146, 179, 182 and 183 in HA gene of the H9N2 AIV in China from 1994 to 2019. All H9 HA amino acid sequences of China isolates (as of November 2019) were downloaded from GISAID (https://www.gisaid.org) and aligned using MAFFT. Excluding homologous and duplicate sequences, the prevalence of amino acids at each antigenic residue was then assessed

To better understand the evolutionary context of the antigenic variation of H9N2 viruses, genetic mutations at the HA positions of 127, 135, 145, 146, 179, 182 and 189 were analyzed (Fig. 6). We used the GISAD database to collect a total of 7098 HA sequences from all H9N2 chicken isolates in China from 1994 to 2019. Excluding duplicate sequences, a total of 6436 sequences were used for data analysis. We found a gradual increase in the amount of the S127D, D135G, T146R, T182R and N183D mutations occurred in China starting in 2012; however, these novel substitutions did not completely replace the previous residues. Soon after, S127D, D135G, T146R and T182R mutations tended to decrease after 2016 and co-circulated with strains harboring the original residues. Notably, the amino acid at site 145 was quite conserved in the H9N2 HA up until the end of November 2019, but the FJ/20 strain tested in this study had a T145N mutation, which may be partly responsible for the antigenic drift of the immune escape variant. In this study, the major substitutions that were responsible for the FJ/20 antigenic variation were mapped to 127D, 135G, 145N and 146R (DGNR), while the vaccine strain AH/17 contained 127S, 135D, 145T and 146Q (SDTQ), suggesting that these four mutations may reduce the immunization efficacy of the current vaccine and lead to more FJ/20-like outbreaks in China.

**Fig. 7:**
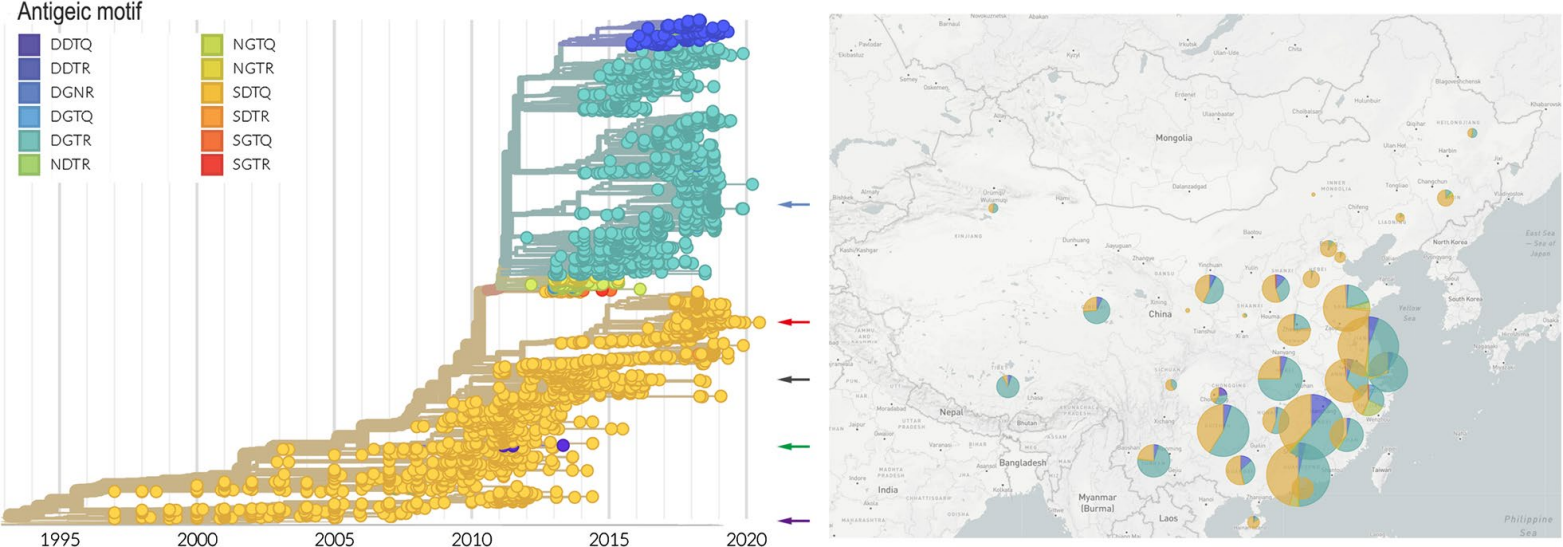
Phylogenetic overview of H9N2 HA in China from1994 to 2020. Left, shows a time-scaled phylogeny of H9N2 HA sequences sampled in China up to the end of June 2020, colored by the motif type in sites 127,135,145 and 146 of HA. The approximate position of vaccine strains is marked with different colored arrows (purple: A/chicken/Shandong/6/1996 (SD/96), green: A/chicken/Guangxi/55/2005 (GX/05), black: A/chicken/Jiangsu/WJ57/2012 (JS/12), red: A/chicken/Anhui/LH99/2017 (AH/17), blue: A/chicken/Fujian/11/2020). Right, the proportion of those same HA sequences containing the different types of motifs in different regions of China. Tree and visualization were generated using the Nextstrain platform (see Methods). Map data copyright Google, INEGI (2021)

Next, a phylogenetic analysis was performed using the 6454 sequences of avian H9N2 in China from 1994 to 2020 to examine whether amino acids at four positions (127, 135, 145 and 146) occurred simultaneously at fixed patterns and whether they were associated with genetic clades (Fig. 7). Herein, we called the combination of amino acids in these four positions as an antigenic motif. More than 60 antigenic motifs at these four positions were identified in all 6436 HA genes, and we selected the 12 most prevalent motifs for phylogenetic analysis. The recombination of four residues (127, 135, 145 and 146) mainly appears as the SDTQ and DGTR motifs from 1994, as shown in Fig. 7. After 2010, H9N2 isolates containing the NDTR, NGTQ, SGTR, SDTR and DGTR antigenic motifs rapidly emerged. Then, viruses containing the DGTR motif circulated simultaneously with strains including the previous SDTQ motif and increased significantly after 2014. Interestingly, viruses containing DGNR were rare in published sequences and emerged around 2016. In this study, FJ/20 contains DGNR. It is unknown whether viruses containing DGNR will gradually become dominant or co-circulate with other motifs need further ongoing epidemiological surveillance.

## Discussion

Understanding the molecular basis by which circulating H9N2 strains escape from the pre-existing antibodies produced by vaccination is important for virus surveillance and appropriate vaccine selection and development. In this study, the immune escape strain A/chicken/Fujian/11/2020 (FJ/20) was examined using HI assays and antigenic cartography. We assessed the contributions of different AA substitutions in the HA protein to understand the antigenic drift and to improve future vaccine development. Our results showed that the residue substitutions in the positions 127, 135, 145, 146, 179, 182 and 183 were critical for converting the antigenicity of both AH/17 and FJ/20. Most importantly, the combination of 127D, 135G, 145N and 146R (DGNR) could be a major factor of antigenic drift in the immune escape variant FJ/20.

Anti-HA antibodies are able to neutralize influenza virus infectivity. Neutralizing antibodies usually bind to highly exposed epitopes of the HA head domain to inhibit virus-cell binding; as a consequence, substitutions in the HA globular head domain are usually required for the generation of variants with epidemic potential. Therefore, we focused on the 18 amino acids of the head domain that differed between FJ/20 and AH/17. The antigenicity of the chimera_III designs for both AH/17 and FJ/20 viruses indicated that seven AA substitutions—positions 127, 135, 145, 146, 179, 182 and 183—are primarily involved in the detected antigenic drift. While these mutations make the antigenicity of FJ/20_Chim_III similar to AH/17 (Fig. 4B), AH/17_Chim_III still has an antigenic distance of 2.6 AU away from FJ/20. This effect could be attributed to the other 22 different residues in the HA1 regions that remained. It is interesting that six out of seven key amino acid substitutions in chimera_III (positions 127, 145, 146, 179, 182 and 183) are located in the corresponding H3 antigenic sites A and B [36], and that they are all near the receptor binding sites. The five sites (135, 145, 146, 179 and 183) in the chimera_III design were strikingly equivalent to five of the key seven antigenic determination-sites recently identified in seasonal human H3N2 influenza viruses circulating from 1968 to 2003 (145, 155, 156, 189, and 193, H3 numbering) [29]. Our results suggest that the antigenic drift of the H9N2 novel variant FJ/20 is mainly driven by simultaneous amino acid substitutions at antigenic sites adjacent to receptor binding sites. The importance of a small number of amino acid positions immediately adjacent to the receptor binding site in shaping antigenic evolution has also been shown for human influenza (H1, H2, and H3) [19, 37], swine H3 [31], and avian H5 [38] influenza A viruses. In addition, the sites 127 and 179 are located in the 130 loop and 190 helix of the HA receptor binding sites, respectively [19, 39]. This suggested that these two substitutions may also influence receptor-binding avidity of H9N2 viruses and lead to antigenic drift [40]. Since recombinant viruses generated in this study did not introduce any additional N-linked glycosylation motif, another studied mechanism of antigenic drift [41, 42], these substitutions in chimera_III might alter the antigenicity of epitopes directly or influence receptor-binding affinity to diminish antibody neutralization.

Our results indicated that the seven substitutions introduced in the chimera_III design were necessary for the antigenic drift from FJ/20 to AH/17. Among those, the combination of 127D/135G/145N/146R (DGNR) was the most responsible for altering its antigenic properties. For FJ/20, the substitution of two or three sites from the chimera_III design had only minor antigenic effects, but all seven mutations from chimera_III together changed the antigenicity of FJ/20 to closely match the AH/17 cluster (Fig. 5B). However, for AH/17 mutant viruses, obvious antigenic drift occurred whenever two or three amino acids introduced individually or by four or five, and the degree of antigenic drift gradually changed with the increasing number of the mutations (Fig. 5C). Collectively, the reciprocally substituted forms of AH/17 and FJ/20 did not mirror the antigenic impact of the other; residues from FJ/20 could more significantly impact the antigenicity of AH/17, but residues from AH/17 had lesser effects on FJ/20. In addition, due to the residue in position 145 being quite conserved and the FJ/20_Chim_III (contacting127S/135D/145T/146Q/179T/182T/183N mutations) were able to be generated by reverse genetics, the FJ/20_127S/135D/145T/146Q, FJ/20_127S/135D/146Q and FJ/20_127S/135D/179T/182T/183N might require additional compensatory mutations for successful virus rescue, and the similar reason as the failure of generation of viable AH/17_Chim_1 and AH/17_145N/146R. This suggested that viral evolution may have occurred with positive selection of antigenic sites and compensatory mutations. Since the putative antigenic motif mainly appears as SDTQ and DGTR forms, AH/17_127D/135G/146R was generated to evaluate the effect of different motifs on antigen change. AH/17_127D/135G/146R had a 12-fold antigenic cross-reactivity reduction with antiserum of vaccine strain AH/17, and there is only 0.7 AU difference between AH/17_127D/135G/146R and AH/17_127D/135G/145N/146R (Additional file 2: Fig. S2). It is unknown whether DGNR-containing viruses will gradually become dominant or continue co-circulating with other motifs and needs ongoing epidemiological surveillance.

Peacock et al. evaluated the binding effect of H9N2 monoclonal antibody escape mutants on chicken antisera, and found that some mutants tend to show little or no inhibition by polyclonal antiserum from immunized chickens [43]. The mutants identified by monoclonal antibodies are important for understanding H9 antigenic diversity, but some mutations identified by monoclonal antibodies are rare or highly conserved in circulating strains. Although some of the HA sites identified in our study have been reported as antigenic sites, the amino acid residues we found differ significantly from those screened by monoclonal antibodies. In most cases, antigenic drift generally relies upon the accumulation of multiple antigenic- and compensatory mutations [18]. For this reason, the effect of simultaneous amino acid substitutions at several key sites on antigenicity was tested. A comprehensive understanding of the antigenicity of circulating H9N2 strains and the early detection of antigenic variations with epidemic and pandemic potential are key to the continued effectiveness of vaccine strains. In this study, we evaluated the relative contributions of different combinations of amino acid substitutions in the HA globular head domain of the immune escape strain FJ/20 and the vaccine strain AH/17. The antigenic changes were then examined using a panel of specific mono-chicken anti-sera obtained from inactivated mutant virus immunization. Studying FJ/20 is of great importance toward our understanding the antigenic properties of circulating H9N2 strains.

## Conclusions

In conclusion, our study demonstrates that S127D, D135G, T145N and N146R mutations in the HA gene caused significant antigenic change when introduced to AH/17, and that these substitutions could be the most responsible for the antigenic drift seen in FJ/20, the vaccine escape mutant. Failure of vaccine protection in FJ/20-like strains resulted in serious outbreaks in China since 2020. The results of this work will be useful for future surveillance efforts in identifying newly sequenced viruses with high potential for immune evasion. Our study could provide further understanding of the evolution and antigenic diversity of H9N2, and these substitution combinations could be key molecular markers for early warning and control of H9N2 outbreaks.

## Supplementary Information

**Additional file 1: Table S1.** GenBank accession numbers. **Table S2.** The information of mono-specific chicken antisera generated in this study. **Table S3.** The hemagglutinin inhibition assay (HI) of the several current vaccine strains and the circulating Fujian/20 virus.

**Additional file 2: Fig. S1.** The overall three-dimensional (3D) antigenic maps. **Fig. S2.** The antigenic map of AH/17, AH/17_127D/135G, AH/17_127D/135G/146R and AH/20_127D/135G/145N/146R.

## Data Availability

All relevant information is provided in this current manuscript. If required, the data presented in this work can be shared by e-mail.

## Abbreviations

IAVs: Influenza A viruses
HA: Hemagglutinin
HI: Hemagglutination inhibition
mAbs: Monoclonal antibodies
