# Supplementary material for: The molecular determinants of antigenic drift in a novel avian influenza A (H9N2) variant virus
Source: Virol J. 2022 Feb 5;19:26. doi: 10.1186/s12985-022-01755-9 (PMC8817646; doi:10.1186/s12985-022-01755-9)
Supplement: Supplementary file 1 — Additional file 1: Table S1. GenBank accession numbers. Table S2. The information of mono-specific chicken antisera generated in this study. Table S3. The hemagglutinin inhibition assay (HI) of the several current vaccine strains and the circulating Fujian/20 virus. [file 12985_2022_1755_MOESM1_ESM.docx]

**Table S1.** GenBank accession numbers

| GenBank accession number | strain name |
| --- | --- |
| MK553631.1 | A/chicken/Jiangxi/X2362/2018 |
| [MK553652.1](https://www.ncbi.nlm.nih.gov/nuccore/MK553652.1) | A/chicken/Jiangxi/X2411/2018 |
| MK553655.1 | A/Duck/Jiangxi/X2437/2018 |
| MK553470.1 | A/chicken/shanghai/s2058/2018 |
| MK553629.1 | A/chicken/Jiangxi/X2359/2018 |
| MK553633.1 | A/chicken/Jiangxi/X2368/2018 |
| MK553627.1 | A/chicken/Jiangxi/X2357/2018 |
| MK552429.1 | A/chicken/Guangdong/G1425/2018 |
| MK552756.1 | A/chicken/Guizhou/Q1400/2018 |
| MK553843.1 | A/chicken/Guangdong/G2106/2017 |
| MK552938.1 | A/chicken/Jiangxi/X1430/2018 |
| MK554395.1 | A/chicken/Jiangxi/X2355/2017 |
| MH489443.1 | A/chicken/Anhui/LH66/2017 |
| MK552946.1 | A/chicken/Jiangxi/X1440/2018 |
| MK554246.1 | A/chicken/Shanghai/S2269/2017 |
| MK553203.1 | A/chicken/Jiangsu/J2177/2018 |
| MH489507.1 | A/chicken/Anhui/AH480/2017 |
| MK552849.1 | A/chicken/Jiangxi/X1107/2018 |
| MK552873.1 | A/chicken/Jiangxi/X1277/2018 |
| MK553993.1 | A/chicken/Jiangsu/J2171/2017 |
| MK554250.1 | A/chicken/Jiangxi/X2016/2017 |
| MK554091.1 | A/chicken/Ningxia/NX2130/2017 |
| MH489451.1 | A/chicken/Anhui/LH99/2017 |
| KJ000710.1 | A/chicken/Jiangsu/WJ57/2012 |
| EU086245.1 | A/chicken/Guangxi/55/2005 |
| JF715024.1 | A/chicken/Guangdong/FZH/2011 |
| KP865959.1 | A/chicken/Shanghai/B469/2011 |
| AF156376.1 | A/duck/Hong Kong/Y280/97 |
| AF508570.1 | A/chicken/Shandong/6/96 |
| KF188366.1 | A/chicken/Hong Kong/G9/1997 |
| KF188294.1 | A/chicken/Beijing/1/1994 |
| AF384557.1 | A/chicken/Guangdong/SS/94 |
| AF461517.1 | A/chicken/Henan/2/98 |
| AY743216.1 | A/chicken/Shanghai/F/98 |
| KP693788.1 | A/chicken/Jiangsu/458/2013 |
| AF156378.1 | A/Quail/Hong Kong/G1/97 |
| AF156384.1 | A/Chicken/Korea/38349-p96323/96 |
| AF156377.1 | A/duck/Hong Kong/Y439/97 |
| AF156388.1 | A/Quail/Arkansas/29209-1/93 |
| AF156386.1 | A/Shorebird/Delaware/9/96 |
| DQ064366.1 | A/chicken/Heilongjiang/35/00 |
| CY130054.1 | A/turkey/Wisconsin/1/1966 |

**Table S2.** The information of mono-specific chicken antisera generated in this study.

| Strain name | Type | HI titer |
| --- | --- | --- |
| A/chicken/Henan/HP/1998 (HN/98) | Wild type | 8.00±1.00 |
| A/chicken/Shandong/6/96(SD/96) | Wild type | 10.67±0.58 |
| A/chicken/Guangxi/55/2005(GX/05) | Wild type | 10.50±0.71 |
| A/chicken/Jiangsu/WJ57/2012(JS/12) | Wild type | 10.67±0.58 |
| A/chicken/Jiangsu/325/2018 (JS/18) | Wild type | 9.00±1.41 |
| A/chicken/Anhui/LH99/2017(AH/17) | Rescued | 10.67±0.58 |
| A/chicken/Fujian/11/2020(FJ/20) | Rescued | 10.33±0.58 |
| AH/17_Chim_II | Rescued | 10.33±0.58 |
| AH/17_Chim_III | Rescued | 10.00±0.00 |
| AH/17_Chim_IV | Rescued | 10.33±0.58 |
| AH/17_127D/135G | Rescued | 8.50±0.71 |
| AH/17_179D/182R/183T | Rescued | 8.50±0.71 |
| AH/17_127D/135G/145N/146R | Rescued | 7.50±0.71 |
| AH/17_127D/135G/179D/182R/183T | Rescued | 10.00±0.00 |
| AH/17_145N/146R/179D/182R/183T | Rescued | 7.50±0.71 |
| FJ/20_Chim_I | Rescued | 10.00±0.00 |
| FJ/20_Chim_II | Rescued | 9.00±0.00 |
| FJ/20_Chim_III | Rescued | 9.67±0.58 |
| FJ/20_Chim_IV | Rescued | 9.00±0.00 |
| FJ/20_127S/135D | Rescued | 10.00±0.00 |
| FJ/20_145T/146Q | Rescued | 8.50±0.71 |
| FJ/20_179T/182T/183N | Rescued | 7.50±0.71 |
| FJ/20_145T/146Q/179T/182T/183N | Rescued | 9.00±0.00 |

**Table S3.** The hemagglutinin inhibition assay (HI) of the several current vaccine strains and the circulating Fujian/20 virus.

| Virus | HI titer^a^ | | | | |
| --- | --- | --- | --- | --- | --- |
|  | Anti-A/Anhui/LH99/2017 | Anti-A/Fujian/11/2020 | Anti-A/chicken/Shandong/6/1996 | Anti- A/chicken/Guangxi/05/2005 | Anti- A/chicken/Jiangsu/WJ57/2012 |
| A/chicken/Anhui/LH99/2017(AH/17) | 2048 | 128 | 1024 | 1024 | 2048 |
| A/chicken/Fujian/11/2020(FJ/20) | **128** | **2048** | **256** | **256** | **128** |
| A/chicken/Shandong/6/1996(SD/96) | 256 | 16 | 2048 | 256 | 512 |
| A/chicken/Guangxi/05/2005(GX/05) | 512 | 64 | 2048 | 1024 | 1024 |
| A/chicken/Jiangsu/WJ57/2012(JS/12) | 1024 | 256 | 1024 | 2048 | 2048 |
