# Supplementary material for: The molecular determinants of antigenic drift in a novel avian influenza A (H9N2) variant virus
Source: Virol J. 2022 Feb 5;19:26. doi: 10.1186/s12985-022-01755-9 (PMC8817646; doi:10.1186/s12985-022-01755-9)

Fig.S1 The overall three-dimensional (3D) antigenic maps.

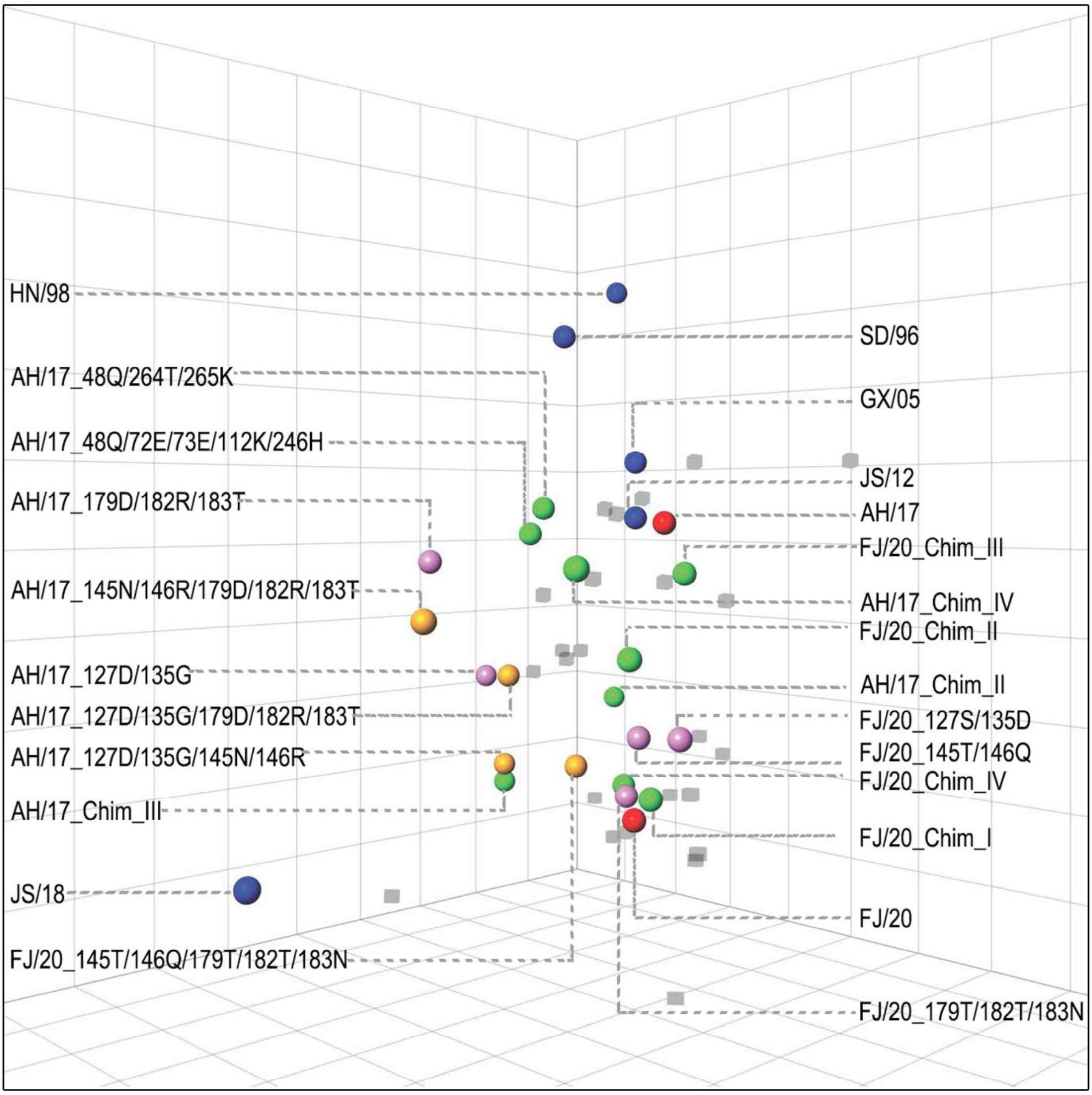

Fig.S2 The antigenic map of AH/17, AH/17_127D/135G, AH/17_127D/135G/146R and AH/20_127D/135G/145N/146R

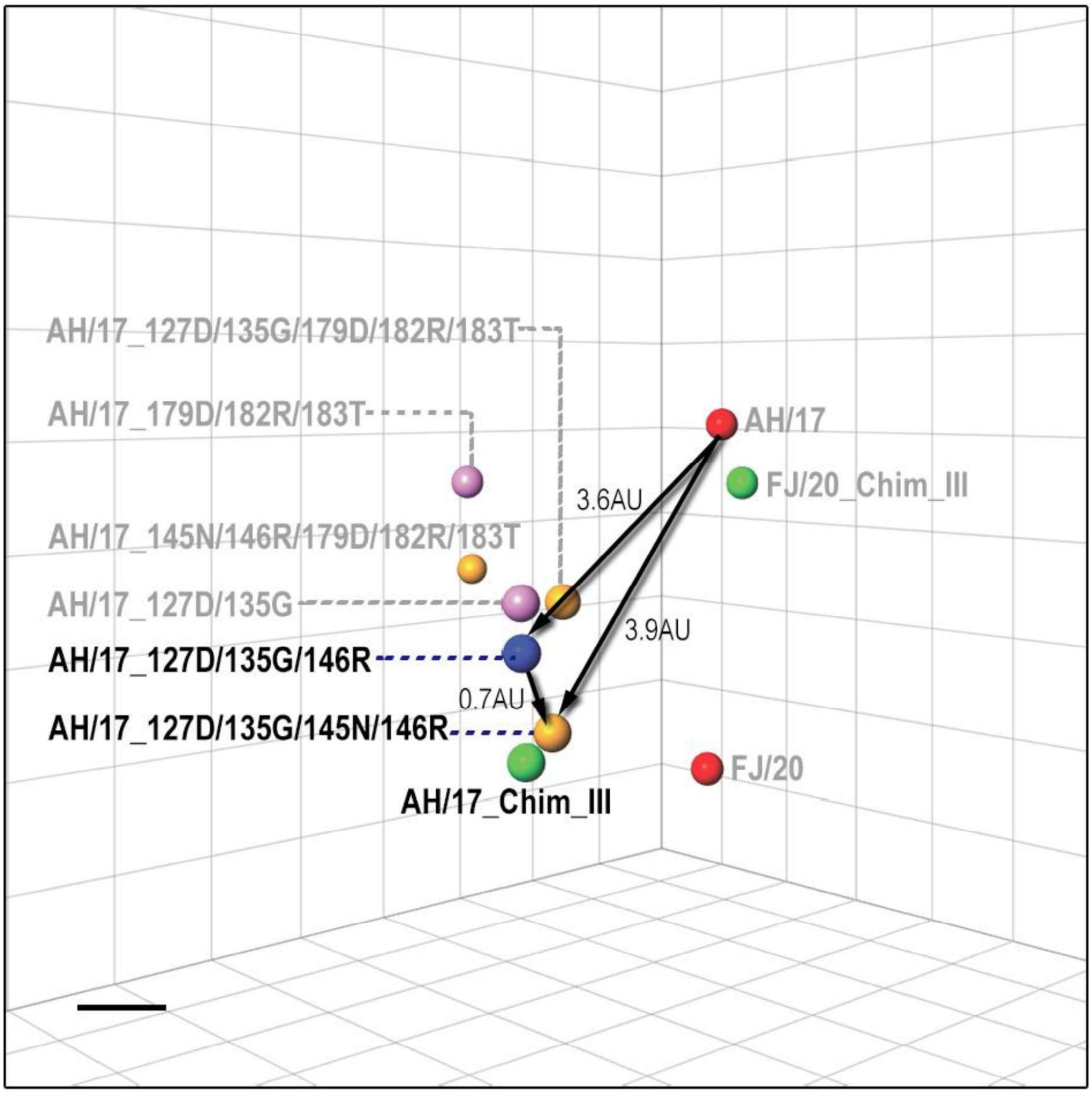

Supplement: Supplementary file 2 — Additional file 2: Fig. S1. The overall three-dimensional (3D) antigenic maps. Fig. S2. The antigenic map of AH/17, AH/17_127D/135G, AH/17_127D/135G/146R and AH/20_127D/135G/145N/146R. [file 12985_2022_1755_MOESM2_ESM.pdf]
